# The epigenetic regulatory effect of histone acetylation and deacetylation on skeletal muscle metabolism-a review

**DOI:** 10.3389/fphys.2023.1267456

**Published:** 2023-12-08

**Authors:** Junjie Xu, Chenglong Li, Xiaolong Kang

**Affiliations:** College of Animal Science and Technology, Ningxia University, Yinchuan, China

**Keywords:** skeletal muscle, histone acetylation, deacetylation, epigenetic modification, muscle metabolism

## Abstract

Skeletal muscles, the largest organ responsible for energy metabolism in most mammals, play a vital role in maintaining the body’s homeostasis. Epigenetic modification, specifically histone acetylation, serves as a crucial regulatory mechanism influencing the physiological processes and metabolic patterns within skeletal muscle metabolism. The intricate process of histone acetylation modification involves coordinated control of histone acetyltransferase and deacetylase levels, dynamically modulating histone acetylation levels, and precisely regulating the expression of genes associated with skeletal muscle metabolism. Consequently, this comprehensive review aims to elucidate the epigenetic regulatory impact of histone acetylation modification on skeletal muscle metabolism, providing invaluable insights into the intricate molecular mechanisms governing epigenetic modifications in skeletal muscle metabolism.

## 1 Introduction

Skeletal muscle, the largest metabolic organ in mammals, accounts for 40%–60% of an animal’s body weight. It possesses a high capacity for substrate oxidation and energy storage, playing a crucial role in the body’s basal metabolic rate, systemic lipid metabolism, and maintenance of blood glucose levels (Egan and Zierath, 2013). Unfortunately, skeletal muscle metabolic disorder is a leading cause of various abnormal function in muscle and chronic diseases (Moresi et al., 2015). Numerous studies have indicated that histone acetylation modifications are involved in several physiological processes related to skeletal muscle. These processes include the skeletal muscle cell cycle, muscle fiber type conversion, muscle atrophy, insulin sensitivity, exercise capacity, and endurance. These modifications occur through the regulation of enzymes responsible for histone acetylation (Tian et al., 2020). To gain a better understanding of the physiological role of skeletal muscle and the molecular regulation of histone acetylation modifications, this review aims to outline the molecular mechanisms by which histone acetylation modifications regulate skeletal muscle metabolism. Additionally, it summarizes the epigenetic effects of histone acetylation and deacetylation modifications on skeletal muscle-related phenotypes. By exploring these epigenetic modification mechanisms associated with muscle development, we can enhance our understanding of the intricate processes involved.

## 2 Histone acetylation

**FIGURE 1:**
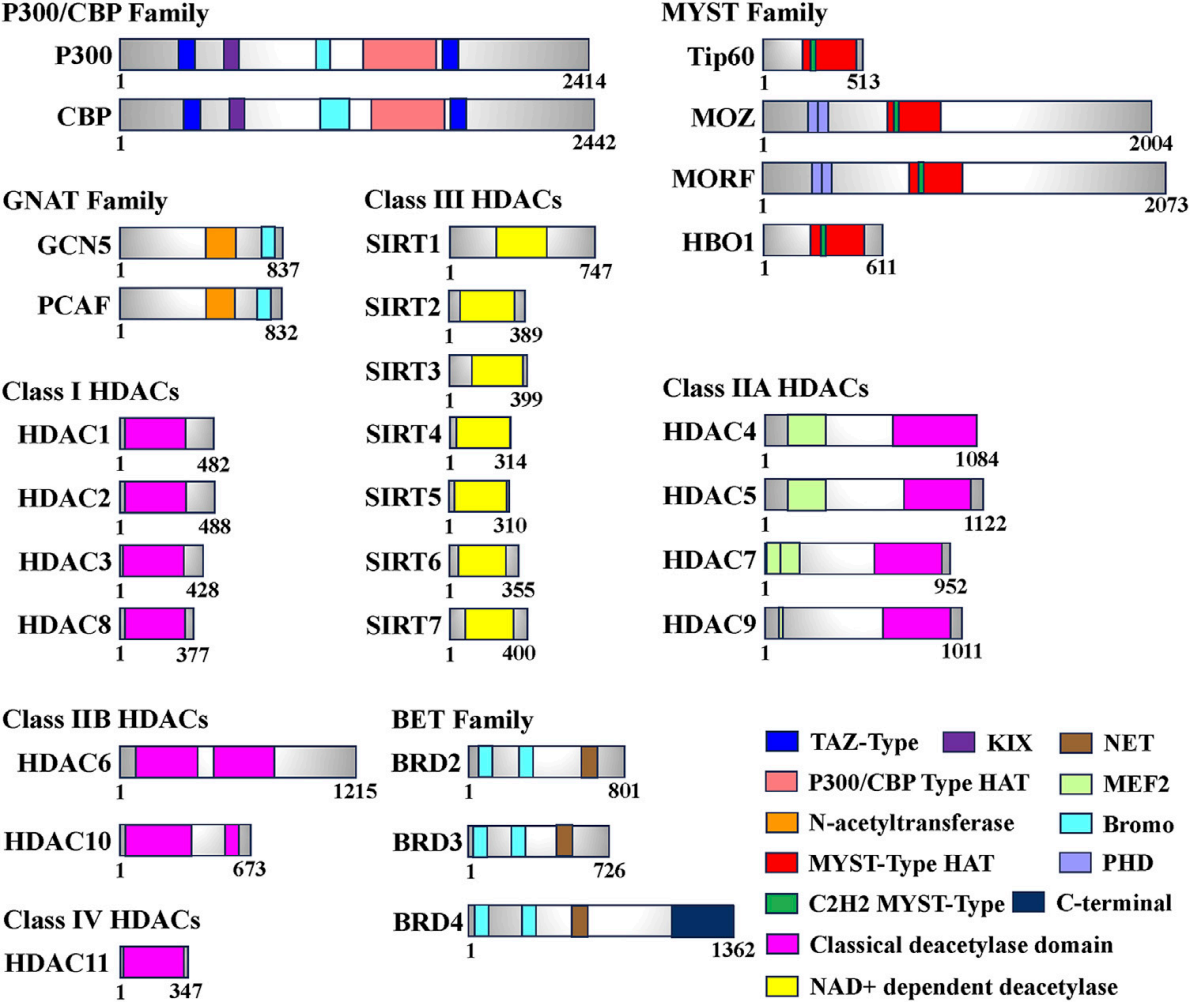
Domain of human histone acetyltransferases (HATs), histone deacetylases (HDACs) and bromodomain and extra-terminal (BET). The total number of amino acids for each enzyme or protein is displayed on the most-right side. In this figure, only the longest isomer of each enzyme or protein is selected, and different colors represent different functional domains.

Histone acetylation modification represents a significant form of post-translational modification (PTM) that affects proteins. It primarily occurs through reversible modifications mediated by histone acetyltransferase (HAT) and histone deacetylase (HDAC) enzymes, targeting various subunits of histones. This dynamic process tightly regulates the levels of histone acetylation. Proteins specifically designed to recognize the acetylation modification state, known as histone acetylation recognition proteins, play a crucial role in recruiting transcriptional regulatory complexes onto chromatin. This recruitment process ultimately governs gene expression (Wernersson et al., 2022). Based on their involvement in the acetylation process, enzymes or proteins related to histone modifications can be further classified into three main categories: Writers (HATs), Erasers (HDACs), and Readers (Histone Acetylation Readers). These classifications are based on the roles these enzymes or proteins play in catalyzing, removing, or recognizing histone acetylation, respectively (Zhao et al., 2018) (Table 1; Figure 1).

**TABLE 1:** Overview of histone acetylation modifiers.

Modification	Modifier	Sequence identity	Subcellular location	Skeletal muscle expression	Function
Acetylation	GCN5 Family (GCN5, PCAF)	71.27%	N/N, C	?/++	Histone and non-histone lysine acetylation Howlett and McGee (2016)
	MYST Family (Tip60, MOZ, MORF, HBO1)	36.70%–60.19%	N, C/N/N/N	++/++/++/++	
	P300/CBP Family (P300, CBP)	63.66%	N, C/N, C	+/+	
	Others (TFIIIC, CLOCK)	19.05%	N/N, C	?/++	
Deacetylation	Class I HDACs (HDAC1, 2, 3, 8)	40.54%–85.06%	N/N, C/N, C/N, C	−/+++/+/++	Zn^2+^ dependent deacetylation of histone and non-histone proteins Beharry et al. (2014)
	Class IIA HDACs (HDAC4, 5, 7, 9)	48.24%–61.60%	N, C/N, C/N, C/N	++/+/?/++	Corepressor recruitment Luo et al. (2019)
	Class IIB HDACs (HDAC6, 10)	41.27%	N, C, P, CS, CP/N, C	−/++	Zn^2+^ dependent deacetylation, primarily of non-histone proteins Kumar and Datta (2022)
	Class III HDACs (SIRT1-7)	20.71%–47.16%	N, C, M/N, C, MB, P, CP/MM/MM/N, C, M/N, ER/N, C	+/++/++/++/++/+++/++	NAD^+^ dependent deacetylation, primarily of non-histone proteins Lee et al. (2023)
	Class IV HDACs (HDAC11)	—	N	?	Zn^2+^ dependentdeacetylation of histone and non-histone proteins Liu et al. (2020)

Note: The Sequence Identity comes from Cluster Omega; Subcellular localization originates from UniProt. expression of modifying enzymes or factors in skeletal muscle comes from the human protein atlas, - no expression; + low expression; ++ medium expression; +++ high expression; ? unknown for each modifier listed. Abbreviation: C, cytoplasm; CP, cell projection; CS, cytoskeleton; ER, endoplasmic reticulum; M, mitochondrion; MB, midbody; MM, mitochondrion matrix; N, nucleus; P, Perikaryon.

### 2.1 Histone acetyltransferase

Histone acetyltransferases (HATs) play a critical role in the process of acetylation by modifying acetyl groups onto lysine residues of both histone and non-histone proteins, including transcription factors. This modification facilitates the dissociation of DNA from nucleosome octamers, leading to the loosening of the nucleosome’s spatial structure. Consequently, this creates an open chromatin conformation, providing a binding site for trans-acting factors such as transcription factors to specifically interact with gene promoter regions and activate gene transcription (Wapenaar and Dekker, 2016).

HATs can be classified into two types, A and B, based on their subcellular localization and substrate specificity. Type A HATs predominantly reside in the nucleus and modify histones associated with chromatin, as well as acetylate non-histone proteins. They primarily contribute to gene transcription. In contrast, type B HATs are found in both the cytoplasm and nucleus, acetylating free histones in the cytoplasm and facilitating their translocation into the nucleus. Studies have shown that A-type HATs play a major role in regulating gene expression (Lee and Workman, 2007; Parthun, 2007). To date, more than 20 types of HATs have been identified, categorized into various families based on their structural domains. Notable families include the P300/CBP family (e.g., P300 and CBP), the GNAT family (e.g., Gcn5 and PCAF), and the MYST family (e.g., Tip60, MOZ, MORF, HBO1). Additionally, certain transcription factors, such as TFIIIC (a universal transcription factor of RNA polymerase III) and CLOCK (an epigenetic clock regulating circadian rhythm in skeletal muscle), are also considered HATs (Howlett and McGee, 2016) (Table1).

**FIGURE 2:**
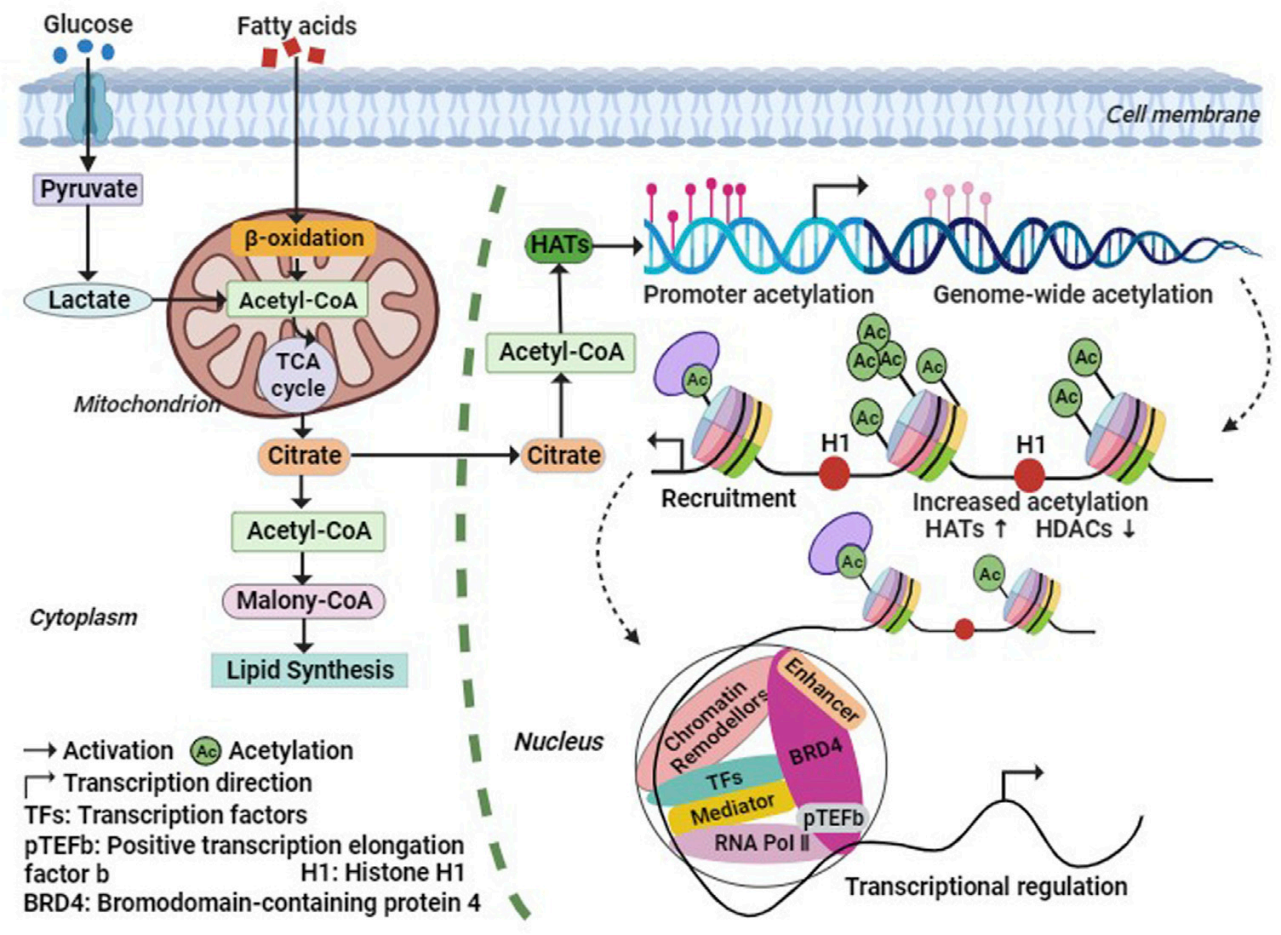
Metabolic regulation processes of histone acetylation. The Acetyl-CoA required for histone acetylation modification, is generated by fatty acids β-Oxidation and Oxidative decarboxylation of Pyruvic acid, and the increase of Acetyl-CoA level will promote the increase of HATs activity. Thus, HATs acetylate histones or non-histones, thereby increasing the transcription level of related genes. Abbreviation: Ac, Acetylation; BRD4, bromodomain containing 4; H1, histone 1; HATs, histone acetyltransferases; HDACs, histone deacetylases; pTEFb, positive Transcription Elongation Factor b; RNA Pol Ⅱ, RNA polymerase Ⅱ; TFs, Transcription Factors.

During histone acetylation modification, the acetyl group is primarily derived from acetyl-CoA, which is generated through fatty acid β-oxidation and oxidative decarboxylation of pyruvate by the pyruvate dehydrogenase complex under aerobic conditions (Su et al., 2016). Phosphorylation of acetyl-CoA promotes histone acetylation, and elevated levels of acetyl-CoA enhance histone acetylation. Conversely, low levels of acetyl-CoA reduce the activity of HATs. Acetyl-CoA serves as a crucial intermediate metabolite in energy metabolism and acts as a hub for glucose, lipid, and protein metabolism (Figure 2). It plays an essential role in maintaining organismal stability (He et al., 2023). Studies have demonstrated that insufficient acetyl-CoA levels result in abnormal intermediate nuclei and mitochondria in mouse skeletal muscle fibers, reduced levels of fully assembled complex I and ATP in the electron transport chain, increased markers of oxidative stress, and significantly impaired exercise capacity and endurance (Corbin et al., 2017). These findings suggest that acetyl-CoA may regulate chromatin dynamics, participate in histone acetylation modifications, indirectly modulate gene expression, and subsequently impact skeletal muscle metabolism.

### 2.2 Histone deacetylase

Histone deacetylases (HDACs) function by catalyzing the removal of acetyl groups from lysine residues on both histone and non-histone proteins, thereby exerting an opposite effect to histone acetyltransferases (Bannister and Kouzarides, 2011). Eukaryotic HDACs can be classified into four classes based on their homology with the three HDACs found in *Saccharomyces cerevisiae* (Rpd3, Hdal, and Sir2) (Gregoretti et al., 2004). Class I HDACs, which share structural similarities with yeast Rpd3, include HDAC1, HDAC2, HDAC3, and HDAC8. Class II HDACs exhibit catalytic structures akin to yeast Hdal and can be further divided into two subclasses, A and B. Class IIA comprises transcriptional co-repressors such as HDAC4, HDAC5, HDAC7, and HDAC9. Class IIB includes HDAC6 and HDAC10. Class III HDACs are homologous to Sir2 in yeast and consist of seven species identified in human cells, known as SIRT1-7 (Seto and Yoshida, 2014). In contrast, Class IV HDACs solely consist of HDAC11, which exhibits sequence similarity in its core catalytic region to both Class I and Class II HDACs. Remarkably, HDAC11 has the smallest molecular weight among known HDACs, measuring only 39 kDa (Thangapandian et al., 2012) (Table 1; Figure 1).

### 2.3 Histone acetylation reader

The bromodomain (BRD) is a highly conserved protein structural domain that specifically recognizes histone acetylation modifications. Dysregulation of the BRD domain has been closely associated with numerous diseases. Based on sequence similarity, proteins containing the BRD domain can be classified into nine families, with the Bromo and Extra-Terminal (BET) family being the most extensively studied (Figure 1). Among the BET family members, Bromodomain-containing 4 (BRD4) plays a crucial role in transcription, DNA replication, and damage repair (Liang et al., 2021). BRD4 has a wide range of substrates and exhibits varying affinities for different sequences. The Bromodomain 1 (BD1) of BRD4 specifically recognizes polyacetylated modifications of histone 4 (H4), with a significantly higher affinity for tetraacetylation compared to monoacetylation. On the other hand, Bromodomain 2 (BD2) can bind to di- or triacetylated modifications and exhibits much lower affinity for tetraacetylation of H4 when compared to BD1 (Slaughter et al., 2021). Additionally, BRD4 can interact with non-histone proteins, such as positive transcription elongation factor b (P-TEFb), thereby promoting RNA polymerase II phosphorylation and facilitating transcription elongation (Figures 1, 2). BRD4 also recognizes the acetylated transcription factor RelA of the NF-κB family (NF-κB p65), regulating the expression of downstream target genes involved in the inflammatory response, including the inflammatory gene IL-6 (Liang et al., 2021; Liu et al., 2022). Moreover, BRD4 is essential for myogenic differentiation. During the differentiation of mouse C2C12 myoblasts, BRD4 selectively binds to the promoter region of the myogenin gene (Myog), coinciding with elevated levels of H3K27ac, a marker of histone acetylation. Conversely, downregulation of BRD3 levels enhances myogenic differentiation, and treatment with JQ1, a BET family inhibitor, produces the opposite effect, highlighting the crucial role of BET proteins in the regulation of skeletal myogenesis (Roberts et al., 2017). These findings demonstrate that histone acetylation recognition proteins can identify the acetylation state and engage with chromatin, thereby remodeling chromatin conformation and regulating skeletal muscle cell differentiation.

## 3 Histone acetylation and skeletal muscle metabolism

### 3.1 Histone acetyltransferases and skeletal muscle metabolism

#### 3.1.1 P300/CBP family

The P300/CBP family primarily consists of P300 and cAMP-response element-binding protein-binding protein (CBP). P300 is a large molecular protein with a size of approximately 300 kDa. It plays a vital role in various cellular processes, including cell cycle regulation, proliferation, differentiation, apoptosis, and the modulation of autophagy and glycolipid metabolism. Alterations in P300/CBP are closely associated with specific cancers and other human diseases. Functional compensatory mechanisms exist between P300 and CBP, as demonstrated by studies showing that double knockdown of P300 and CBP leads to rapid changes in gene expression patterns related to skeletal muscle function. This results in loss of contractile function and ultimately leads to the death of experimental mice within 1 week. However, knockdown of P300 or CBP alone does not result in lethal phenotypes. While skeletal muscle function is partially impaired, it remains sufficient to maintain normal physiological activity (LaBarge et al., 2016). These findings suggest that a single allele of CBP or P300 is capable of maintaining normal skeletal muscle function (Svensson et al., 2020a).

**FIGURE 3:**
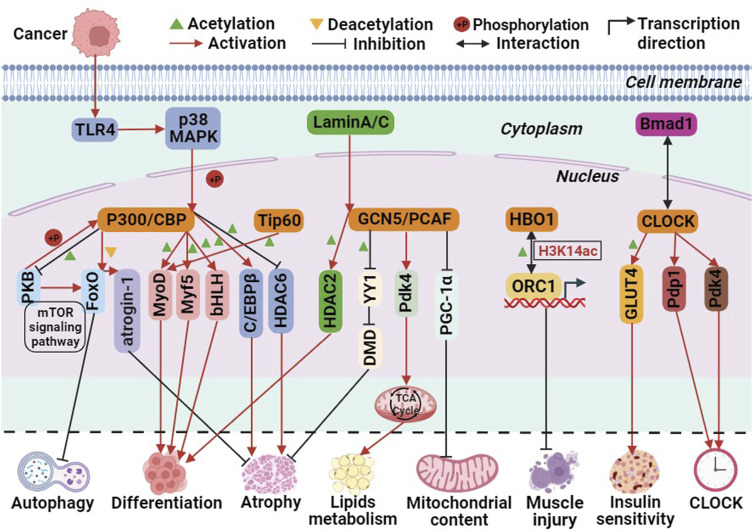
The molecular role of histone acetyltransferases (HATs) involved in biological processes related to skeletal muscle metabolism. Among them, green triangle is acetylation, yellow triangle is deacetylation, + P is phosphorylation, red arrows is activation, black prohibitory symbol is inhibition, black bidirectional arrow is interactions, and black folded arrow is transcription start sites.

Currently, three pathways have been identified in which P300 is involved in biological processes. First, P300 acts as a histone acetyltransferase, modifying histones through acetylation to promote transcription. Second, it acetylates non-histone proteins, such as transcription factors, thereby enhancing their transcriptional activity. Third, it functions as a transcriptional co-activator, recruiting specific transcription factors to the promoter region of genes to activate transcription (Cheng et al., 2019). These pathways are also implicated in P300-mediated skeletal muscle cell differentiation. For instance, P300 mediates skeletal muscle cell transcription and terminal differentiation by acting upstream of the myogenic regulatory factors MyoD and Myf5 (Roth et al., 2003). Moreover, P300 activates target genes by interacting with the basic helix-loop-helix (bHLH) structural domain of tissue-specific transcription factors, thereby regulating skeletal muscle cell differentiation (Eckner et al., 1996). Additionally, the Akt/protein kinase B (PKB) pathway acts as a positive regulator of P300. PKB phosphorylates and activates P300, thereby mediating myoblast differentiation (Chen et al., 2015). In human and mouse skeletal muscle cells, P300/CBP interacts with PKB to regulate skeletal muscle insulin sensitivity. PKB phosphorylates and activates P300/CBP, while P300/CBP acetylates and inactivates PKB. This interaction forms the PKB-P300/CBP axis, influencing insulin signaling, glucose transporter 4 (GLUT4) transport, and metabolism processes (Martins et al., 2019; Martins et al., 2022). The mechanism by which P300 mediates skeletal muscle atrophy varies between healthy and diseased individuals. In healthy individuals, P300/CBP-mediated skeletal muscle atrophy is primarily mediated through the FoxO signaling pathway. Decreased P300/CBP activity in rat soleus muscle and mouse C2C12 myogenic cells leads to increased FoxO reporter gene activity, resulting in the transcriptional activation of its target gene, atrogin-1 (Senf et al., 2011). In type 2 diabetes-induced skeletal muscle atrophy, P300 is overactivated and acetylates PKB and its main downstream effectors, mTOR and FoxO, effectively blocking autophagy in skeletal muscle cells. Additionally, acetylated insulin receptor substrate inhibits its binding with the insulin receptor, thereby disrupting insulin signaling and causing skeletal muscle atrophy (Fan et al., 2020). In cancer-induced skeletal muscle atrophy, overexpression of Toll-like receptor 4 (TLR4) leads to the phosphorylation of P300 by p38β MAPK, a central regulator of skeletal muscle atrophy. This phosphorylation stimulates C/EBPβ acetylation, resulting in muscle atrophy (Sin et al., 2021). Rhabdomyosarcoma (RMS) is the most common soft tissue sarcoma in children caused by impaired myogenic differentiation, with two main subtypes: ‘embryonal’ RMS (ERMS) and ‘alveolar’ RMS (ARMS) (Skapek et al., 2019). Garcinol and Anacardic Acid, natural inhibitors of histone acetyltransferases, may inhibit RMS cells growth and proliferation via P300/CBP(Tomasiak et al., 2023). Furthermore, glucocorticoids upregulate P300 expression in skeletal muscle and decrease HDAC6 activity, contributing to muscle atrophy (Alamdari et al., 2010). These findings indicate that the P300/CBP family regulates skeletal muscle cell differentiation, autophagy, atrophy, and insulin signaling through the acetylation of histones and non-histones or as transcriptional co-activators (Figure 3).

#### 3.1.2 GNAT family

The GCN5-Related N-acetyltransferases (GNAT) family primarily consists of general control non-derepressible 5 (GCN5) and P300/CREB binding protein associated factor (PCAF) (Neuwald and Landsman, 1997). The C-terminal region of the GCN5 protein is mainly responsible for recognizing histone acetyl groups, while the N-terminal region recognizes nucleosomes (Figure 1). The HAT structural domain of GCN5 is associated with the recognition of acetyl-CoA (Salah Ud-Din et al., 2016; Albaugh and Denu, 2021). GCN5 utilizes its histone acetyltransferase activity to regulate genes and transcription factors involved in skeletal muscle metabolism. For instance, GCN5 functions as a negative regulator of peroxisome proliferator activator receptor γ-coactivator 1α (PGC-1α), inhibiting its activity and thereby limiting mitochondrial content. However, the homologue PCAF compensates for the absence of GCN5, which explains why knocking down GCN5 alone does not increase skeletal muscle mitochondrial content. GCN5 acetylates and inhibits the transcription factor YY1, a negative regulator of muscle fibers, disrupting the interaction between the zinc finger region of YY1 protein and DNA. This acetylation leads to increased expression of structurally critical proteins in muscle, thereby maintaining the integrity of skeletal muscle cells (Addicks et al., 2022).

Moreover, in mouse skeletal muscle, adaptation to a high-fat diet involves the regulation of the target gene Pdk4 (pyruvate dehydrogenase kinase 4), a key regulator of the tricarboxylic acid cycle, through GCN5(Svensson et al., 2020b; Green et al., 2022). In human skeletal muscle cells, PCAF has been identified as an acetylated histone acetyltransferase (HAT) of HDAC2. The nuclear lamina protein A/C (Lamin A/C), responsible for maintaining structural function and transcriptional regulation in the nucleus, recruits PCAF and HDAC2 to promote myogenic cell differentiation. During this process, PCAF acetylates Lys75 of HDAC2. The expression of mutant forms of Lamin A/C inhibits the translocation of PCAF to the nuclear membrane, thereby impairing myoblast differentiation and leading to Emery-Dreifuss muscular dystrophy (Santi et al., 2020) (Figure 3). These findings suggest that the GNAT family, through the acetylation and activation of HDAC2, can promote myoblast differentiation, highlighting the complex interplay of HATs and HDACs in regulating skeletal muscle metabolism.

#### 3.1.3 MYST family

The MYST family primarily consists of Tip60, MOZ, MORF, HBO1, and MOF. All members of the MYST family share a highly conserved MYST structural domain, which includes a histone acetyltransferase (HAT) functional domain, a zinc finger domain, and an acetyl-CoA binding site. The HAT functional domain is responsible for the binding of acetyl-CoA and the substrate, and the active site lysine is crucial for the self-acetylation activity of HATs (Yuan et al., 2012). This self-acetylation feature distinguishes the MYST family from other HATs (Figure 1). Additionally, some members possess specific structures like the chromodomain (ChD) (Avvakumov and Cote, 2007). HIV Tat-interacting protein of 60 kDa (Tip60) acetylates core histones H2A, H3, H4, and transcription factors, thereby participating in the regulation of transcriptional processes. It plays important roles in transcriptional regulation, signal transduction, DNA damage repair, and other cellular functions (Sun et al., 2010). Tip60 recruits myogenic determinants, such as MyoD, to the myogenin gene promoter and enhances the transcriptional activity of MyoD. This is achieved through physical interactions between Tip60’s ChD and plant homeodomain-linked zinc finger (PHD) structural domains with MyoD. Ectopic expression of Tip60 also enhances the transcriptional activity of myogenic regulatory genes. Conversely, knockdown of Tip60 in mouse C2C12 cells inhibits myogenic cell differentiation (Kim et al., 2011).

Monocytic leukemia zinc finger (MOZ) serves as a binding chaperone of CBP. MOZ and MOZ related factor (MORF) possess two tandem PHD domains (Perez-Campo et al., 2009) (Figure 1). These proteins acetylate histones H3 and H4, including self-acetylation, and function as transcriptional co-activators (Bristow and Shore, 2003; Cai et al., 2010). They also play a key role in the selectivity of core histone H3 and its binding to chromatin (Ali et al., 2012). MOZ and MORF are closely associated with the maintenance of fetal embryonic stem cells and the development of diseases such as tumors (Yang, 2015). However, their role in skeletal muscle remains unclear.

Histone acetyltransferase binding to ORC1 (HBO1) interacts with ORC1, the initiating subunit of DNA replication, and is highly enriched at the transcription start site (TSS) of active genes. HBO1 is a key enzyme for H3K14 acetylation and can act as a coactivator in the regulation of replication initiation. It plays crucial roles in DNA replication, transcription regulation, and other cellular processes (Xiao et al., 2021). Early in the regeneration process of mouse C2C12 myoblasts, the expression of adipocyte differentiation factor 24 (fad24), a positive regulator of adipocytes, and HBO1 is upregulated in response to cardiotoxin-induced muscle damage (Ochiai et al., 2016) (Figure 3).

#### 3.1.4 Others

Transcription factor IIIC (TFIIIC) is a general transcription factor of RNA polymerase III that possesses histone acetyltransferase (HAT) activity. It plays a crucial role in forming a transcription initiation complex by binding to DNA in the promoter region and recruiting the TATA-binding protein (TBP) in the core promoter region, as well as the general transcription factor TFIIIB and RNA polymerase III (Figure 2). TFIIIC exhibits histone acetyltransferase activity and acetylates histones H2A, H3, H4, and nucleosomes (Dumay-Odelot et al., 2007). However, the specific mechanism by which TFIIIC functions in skeletal muscle has yet to be identified.

Skeletal muscle exhibits a highly autonomous circadian rhythm, primarily regulated by a transcription-translation negative feedback loop involving core CLOCK genes. Disruptions in circadian rhythms can negatively impact glucose, lipid, and amino acid metabolism, increasing the risk of metabolic diseases such as obesity and diabetes (Li and Chen, 2020). The CLOCK gene and its associated transcription factors, including brain and muscle ARNT-like protein 1 (Bmal1), form dimers that enhance the HAT activity of CLOCK and the expression of genes related to skeletal muscle metabolism. Knocking down Bmal1 disrupts the circadian rhythm of skeletal muscle metabolism gene expression, leading to reduced insulin sensitivity and glucose uptake (Dyar et al., 2014) (Figure 3).

### 3.2 Histone deacetylases and skeletal muscle metabolism

#### 3.2.1 Class I HDACs

HDACs play a crucial role in the regulation of crosstalk between skeletal muscle and other organs, impacting tissues such as the liver and adipose tissue. They exert their influence by controlling the production of myokines, which are involved in the development of cancer, obesity, diabetes, and cardiovascular diseases, making them essential for overall body metabolism (Renzini et al., 2022).

**FIGURE 4:**
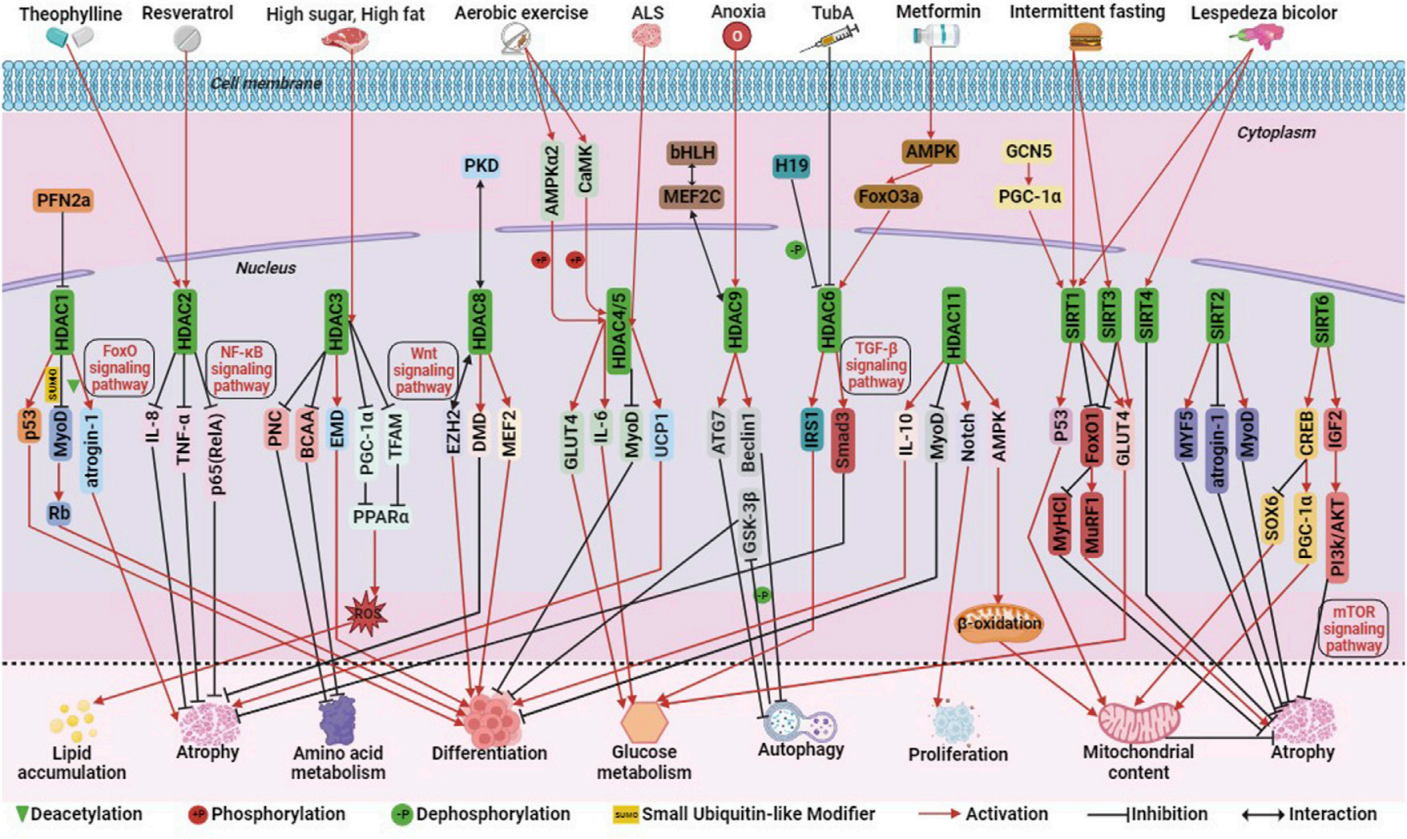
The molecular regulation of histone deacetylases (HDACs) involved in skeletal muscle metabolism. Among them, green triangle is deacetylation, +P is phosphorylation, -P is dephosphorylation, SUMO is ubiquitination-like, red arrow is activation, black prohibitory symbol is inhibition, and black bi-directional arrow is interaction.

Among Class I HDACs (including HDAC1, 2, 3, 8), HDAC1, similar to P300, can mediate skeletal muscle atrophy through the FoxO signaling pathway. Studies have demonstrated that HDAC1 regulates the expression of the muscle atrophy gene atrogin-1 and activates the FoxO signaling pathway, leading to muscle fiber atrophy. Treatment with MS-275, a Class I HDAC inhibitor, significantly alleviates muscle fiber atrophy and contractile dysfunction, highlighting the significant role of HDAC1 in skeletal muscle atrophy (Beharry et al., 2014). During the development of mouse C2C12 myoblasts, the actin monomer-binding protein Profilin 2a (PFN2a) inhibits the nuclear localization and activity of HDAC1. This inhibition enhances the activity of the tumor suppressor protein p53, resulting in the inhibition of C2C12 myoblast proliferation and differentiation and promoting apoptosis (Li et al., 2019). Additionally, the small ubiquitin-like modifier (SUMO) modification of HDAC1 plays a dual role in MyoD signaling. Single SUMO modification promotes the deacetylation of MyoD in undifferentiated cells, while multiple SUMO modifications promote a shift of its binding partner from MyoD to the tumor suppressor protein Rb, thus inducing myoblast differentiation (Joung et al., 2018). HDAC2 ameliorates skeletal muscle dysfunction caused by chronic obstructive pulmonary disease (COPD) primarily through the activation of the NF-κB signaling pathway. Maintaining adequate levels of HDAC2 may serve as a therapeutic target for improving COPD-induced myasthenia. Theophylline, a clinical therapeutic agent for obstructive pulmonary disease, enhances skeletal muscle function by upregulating HDAC2 and reducing the levels of p65 (RelA) in the nuclear transcription factor NF-κB family. It also inhibits the activity of interleukin-8 (IL-8) and tumor necrosis factor TNF-α, further improving muscle function (To et al., 2017; Bin et al., 2019; Li et al., 2021). In addition, the mechanism of action of resveratrol in improving skeletal muscle atrophy and aging is similar to that of theophylline (Sun et al., 2017; Li et al., 2022; Parrella et al., 2022) (Figure 4).

Hyperactivation of HDAC3 in mice fed a high-fat or high-sugar diet inhibits the activity of the transcriptional coactivator PGC-1α and mitochondrial transcription factor A (TFAM), reducing the expression of peroxisome proliferator-activated receptor alpha (PPARα) and key mitochondrial metabolic enzymes. This leads to impaired mitochondrial oxidation, increased production of mitochondrial reactive oxygen species (ROS), and intracellular accumulation of triglycerides (TG). Treatment with MS-275, a class I HDAC inhibitor, reverses these effects, underscoring the critical role of HDAC3 in regulating insulin sensitivity, lipotoxicity, and stress signaling in skeletal muscle cells (Lee et al., 2020). It has been shown that HDAC3 may also serve as a therapeutic target for rhabdomyosarcoma. MS-275 may also be an epigenetic therapeutic drug for RMS. MS-275 reduced the activity of HDAC3 and downregulated the expression of the chromatin remodeling enzyme SMARCA4, which in turn reduced the activity of miR-27a, leading to decreased expression of PAX3:FoxO1, and destabilization of mRNA in ARMS cells (Bharathy et al., 2018). HDAC3 also activates the myotonic dystrophy gene EMD and reduces H4K5ac, a histone acetylation marker, thereby regulating myoblast differentiation (Bossone et al., 2020). Additionally, HDAC3 directly inhibits the catabolism of branched-chain amino acids (BCAAs) and the transcription of purine nucleotide cycle (PNC) genes, thus impacting amino acid metabolism in skeletal muscle (Gong et al., 2018) (Figure 4).

HDAC8 differs from other Class I HDACs in terms of its regulation mode. Phosphorylation at the Ser39 site located on the surface of HDAC8 disrupts its surface structure and inhibits its activity. Conversely, mutation of the Ser39 site to alanine enhances HDAC8 activity (Somoza et al., 2004). HDAC8 acts as a potential feedback factor for PKD phosphorylation, regulating myogenic gene expression and inhibiting PKD phosphorylation in response to stress signals in mouse C2C12 myoblasts (Habibian et al., 2023). HDAC8 also physically interacts with the polycomb protein EZH2 to activate the Wnt signaling pathway, thereby regulating skeletal muscle cell differentiation (Ferrari et al., 2019). Clinical studies have shown that HDAC8 is overactivated in skeletal muscle of patients with Duchenne Muscular Dystrophy (DMD). Treatment of human primary myoblasts with PCI-34051 (an HDAC8 inhibitor) and Givinostat (a broad-spectrum HDAC inhibitor) slows skeletal muscle degeneration and maintains skeletal muscle integrity, highlighting HDAC8 as a potential therapeutic target for DMD (Spreafico et al., 2021). Similar to the action of PCI-34051, sulforaphane (SFN) decreases HDAC8 activity, phosphorylates CREB, acetylates p53 and upregulates their expression, thereby upregulating PGC-1α expression and promoting mitochondrial biogenesis (Yang et al., 2023). These findings demonstrate that Class I HDACs regulate skeletal muscle metabolism through various signaling pathways such as FoxO, NF-κB, and Wnt, emphasizing the complexity of their mechanisms of action (Figure 4).

#### 3.2.2 Class ⅡA HDACs

Class IIA HDACs, including HDAC4, 5, 7, and 9, play important roles in skeletal muscle regulation. HDAC4 is involved in the developmental process of chicken skeletal muscle satellite cells (Zhao et al., 2020). It acts on myosin heavy chain, PGC-1α, and heat shock homolog Hsc70 to maintain skeletal muscle homeostasis (Luo et al., 2019). Similar to P300 and CBP, HDAC4 and HDAC5 exhibit functional compensation. These HDACs are closely associated with exercise-induced alterations in skeletal muscle metabolic patterns. Aerobic exercise activates calcium/calmodulin-dependent protein kinase (CaMK) and adenylate-activated protein kinase α2 (AMPKα2), which phosphorylate and activate HDAC4/5. This leads to their translocation into the cytoplasm, reduced activity in the nucleus, inhibition of MyoD-dependent gene transcription, and impeded differentiation of skeletal myoblasts into myotubes (Yuan et al., 2014; Renzini et al., 2018). Additionally, exercise promotes the dissociation of HDAC4/5 from the GLUT4 promoter and myocyte enhancer factor 2 (MEF2), resulting in increased transcription levels of GLUT4 and interleukin-6 (IL-6), enhanced glucose uptake, and oxidation (Niu et al., 2017; Klymenko et al., 2020). Further studies have shown that the elevation of HDAC5 activity during exercise is not solely dependent on AMPK but also involves the upregulation of muscle-specific ring-finger protein 1 (MuRF1), an E3 ligase targeting HDAC5. MuRF1-mediated ubiquitination leads to HDAC5 degradation by the ubiquitin-proteasome system and its dissociation from MuRF1 and the 20S proteasome (Huang et al., 2022). Elevated levels of PAX3/7-FoxO1 expression in ERMS cells induced expression of the oxidative stress response factor HO-1 and a reduction in reactive oxygen species, leading to inhibition of nuclear localization of HDAC4 and its target miR-206, and thus inhibition of myogenic differentiation (Ciesla et al., 2016). In ARMS cells, PAX3-FOXO1 inhibits IL-24 activity in a HDAC5 dependent manner, thereby promoting cell proliferation, survival, and migration (Lacey et al., 2018). In addition, studies have shown that HDAC4 mediates neural skeletal muscle interactions, and its expression in skeletal muscle is associated with disease severity, which has been proposed as a target for Amyotrophic Lateral Sclerosis (ALS). Patients with amyotrophic lateral sclerosis have high expression of HDAC4 in their skeletal muscles. The absence of HDAC4 can lead to early onset of ALS, weight loss, skeletal muscle atrophy, and lipid metabolism disorders. HDAC4 may act on UCP1 and Runx2 to alleviate the phenotypic symptoms of ALS (Vega et al., 2004; Pigna et al., 2019; Burg et al., 2021) (Figure 4).

HDAC9 is closely associated with skeletal muscle cell differentiation and atrophy. It regulates skeletal muscle cell differentiation through a negative feedback loop, where myocyte enhancer factor 2C (MEF2C) activates HDAC9 gene expression. However, HDAC9 can bind to MEF2 protein and repress its transcriptional activity, thereby inhibiting HDAC9 transcription (Haberland et al., 2007). HDAC9 also plays a role in skeletal muscle atrophy induced by hypoxic conditions. Hypoxia increases HDAC9 levels, and HDAC9 directly binds to the promoter regions of autophagy-related proteins ATG7, Beclin1, and LC3 to inhibit autophagy. This leads to sequential dephosphorylation of glycogen synthase kinase 3β (GSK-3β) and inactivation of the classical Wnt signaling pathway. Consequently, myogenic cell differentiation and multinucleated myotube formation are hindered (Zhang et al., 2019). In summary, the regulatory role of Class IIA HDACs in skeletal muscle metabolism is directly influenced by their nucleocytoplasmic localization. They participate in exercise-induced metabolic adaptations, differentiation processes, and regulation of skeletal muscle homeostasis (Figure 4).

#### 3.2.3 Class IIB HDACs

Class IIB HDACs, including HDAC6 and HDAC10, have distinct characteristics and functions (Figure 1). HDAC6 plays a role in regulating glucose metabolism and muscle atrophy in skeletal muscle through its interaction with the lncRNA H19. The H19-HDAC6-IRS1 axis is important for glucose homeostasis and metabolism (Kumar and Datta, 2022). HDAC6 is also involved in muscle atrophy caused by Metformin, acting through the AMPK-FoxO3a-HDAC6 axis (Ratti et al., 2015; Kang et al., 2022). Inhibition of HDAC6 using TubA improves skeletal muscle disorders in Duchenne Muscular Dystrophy (DMD) by modulating TGF-β signaling (Osseni et al., 2022). In RMS cells, HDAC6 regulates cytoskeletal dynamics through the Rho family GTPase Rac1 to promote tumor cell migration and invasion, and targeting the HDAC6-Rac1 axis may be a therapeutic target for RMS(Pham et al., 2021). HDAC inhibitors may become therapeutic drugs for RMS, such as the novel dual BET/HDAC inhibitor TW09, which mediates cell death through mitochondrial apoptosis in RMS cells (Haydn et al., 2017; Laszig et al., 2020). On the other hand, HDAC10 is implicated in DNA damage repair and immune cell expression. Exercise increases HDAC10 gene expression in human skeletal muscle, impacting skeletal muscle plasticity by regulating metabolic enzymes and chromatin modifying enzymes or transcriptional regulators (Islam et al., 2017; Mina-Paz et al., 2022) (Figure 4). Overall, Class IIB HDACs contribute to the regulation of skeletal muscle fibers through modulation of chromatin spatial conformation, these HDACs impact skeletal muscle plasticity and chromatin structure.

#### 3.2.4 Class Ⅲ HDACs

Class Ⅲ HDACs primarily encompass the SIRT protein family, consisting of SIRT1-7. These proteins interact with various regulatory factors, including p53, FoxO/PGC-1α, NF-κB, and Ku70, to modulate genomic stability, cellular stress response, metabolism, senescence, and apoptosis. SIRT1 plays a significant role in skeletal muscle function, often associated with GCN5, SIRT3, and SIRT4. GCN5 activates PGC-1α, leading to increased expression of crucial genes involved in glycolipid metabolism by augmenting SIRT1 activity. However, overexpression of SIRT1 or knockdown of GCN5 fails to promote exercise-induced metabolic remodeling in mouse skeletal muscle (Svensson et al., 2020b). In the context of diabetic skeletal muscle, Lespedeza bicolor, an agent with antidiabetic properties, has been shown to activate SIRT1, SIRT3, SIRT4, and PGC1α, leading to enhanced mitochondrial biogenesis, inhibition of apoptosis, and improvement in skeletal muscle atrophy (Surinlert et al., 2021; Lee et al., 2023). SIRT3 has also been implicated in mediating insulin sensitivity in rat skeletal muscle through the regulation of GLUT4 (Zhang P. et al., 2022). SIRT1 and SIRT3 are highly expressed in slow twitch muscle fibers, and their activation by intermittent fasting prevents type I myofiber atrophy by inhibiting the transcriptional activity of FoxO1 and FoxO3. This inhibition suppresses the expression of the type I myosin heavy chain gene and prevents atrophy in type I myofibers. Moreover, SIRT1 may synergistically interact with p53, thereby enhancing muscle fatigue resistance during muscle injury repair. Notably, SIRT1 overexpression significantly alleviates muscle pathology associated with Duchenne Muscular Dystrophy (DMD) (Myers et al., 2019). The SIRT3 gene is unique, as it generates three distinct protein isoforms with varying mitochondrial localization efficiency and stability. Overexpression of the SIRT3M3 isoform activates AMPK and PPARδ, promoting a higher proportion of slow twitch muscle fibers. However, this upregulation also increases the levels of the FoxO1 transcription factor and its downstream muscle atrophy gene, MuRF-1, resulting in a 30% reduction in muscle mass (Lin et al., 2014). Additionally, SIRT3 upregulation affects tafazzin gene expression, leading to a decrease in the core phospholipid content of mitochondrial metabolism and alterations in phospholipid and fatty acid composition (Chabi et al., 2018) (Figure 4).

SIRT2 plays a role in skeletal muscle processes such as repair after injury, muscle atrophy, and insulin sensitivity. It positively regulates skeletal muscle cell regeneration by upregulating myogenic regulators (Myf5, MyoD, Myogenin), cell cycle regulators (cyclin D1, CDK2), and downregulating the myasthenic gene atrogin1, thereby promoting anabolic signaling and inhibiting catabolic signaling to improve muscle atrophy following injury (Han et al., 2021; Lee et al., 2022). Conversely, knockdown of SIRT2 improves insulin sensitivity in insulin-resistant skeletal muscle cells (Lantier et al., 2018). SIRT5’s role in skeletal muscle remains unidentified, although it has been implicated in processes related to glioblastoma, melanoma, and acute myeloid leukemia (Bringman-Rodenbarger et al., 2018). The activity of SIRT6 in skeletal muscle metabolism has a dual nature. Increased SIRT6 activity activates AMPK, leading to improved glucose uptake and utilization, thereby maintaining insulin sensitivity (Cui et al., 2017). Furthermore, SIRT6 activates the SIRT6-CREB-Sox6 axis, which promotes the production of slow twitch muscle fibers. SIRT6 upregulates the expression of the slow myofiber activator PGC-1α downstream of CREB, inhibits the activity of the slow twitch muscle inhibitor Sox6, and enhances slow myofiber recruitment. This process increases mitochondrial content and oxidative capacity, resulting in improved exercise endurance (Song et al., 2022). On the other hand, reduced SIRT6 activity significantly upregulates IGF2 expression, overactivates the PI3K/AKT signaling pathway, leading to FoxO inactivation and transcriptional activation of the mTOR signaling pathway. Consequently, protein synthesis is increased, preventing muscle atrophy (Mishra et al., 2022) (Figure 4). SIRT7 maintains genomic and telomere stability, regulates protein homeostasis, mitochondrial function, glucose homeostasis, stem cell activity, and participates in intercellular communication and aging (Lagunas-Rangel, 2022). Elevated SIRT7 activity inhibits apoptosis of myoblasts in hyperglycemic mice (Surinlert et al., 2021). All these indicate that Class III HDACs, along with the GNAT family, contribute to the regulation of myofibers and the remodeling of skeletal muscle metabolic patterns, the specific regulatory mechanisms need time to be revealed and elucidated.

#### 3.2.5 Class Ⅳ HDACs

HDAC11, a latecomer in the field of epigenetics, has emerged as a crucial player in various biological processes, including tumor development, immune dysfunction, barrier function, ischemic damage, lipid metabolism, genomic stability, and cell cycle progression (Liu et al., 2020). During the differentiation of C2C12 myoblasts in mice, HDAC11 exhibits significant activity, and its ectopic expression completely inhibits myoblast differentiation. Similar to HDAC4 and HDAC5, HDAC11 downregulates the transcription of MyoD, thereby impeding myoblast differentiation (Byun et al., 2017). HDAC11 also promotes bovine skeletal muscle satellite cell proliferation through the activation of the Notch signaling pathway (Zhang R. et al., 2022). Interestingly, contrary to SIRT6, inhibiting HDAC11 activity upregulates the expression of IL-10, a factor that enhances myogenic differentiation, facilitating the differentiation of skeletal muscle satellite cells and accelerating muscle regeneration (Nunez-Alvarez et al., 2021). Furthermore, HDAC11 contributes to the production of oxidative myofibrils, increases mitochondrial content, activates the AMP-activated protein kinase-acetyl CoA carboxylase pathway, promotes mitochondrial fatty acid β-oxidation, reduces acylcarnitine levels, and enhances fatigue resistance and muscle strength (Hurtado et al., 2021) (Figure 4). It is important to note that HDAC11 exhibits not only deacetylation activity but also a defatty-acylation activity that surpasses its deacetylation activity by more than 10,000 times (Cao et al., 2019).

## 4 Epigenetic heritability of skeletal muscle metabolism

Epigenetics encompasses heritable phenotypes that are not dependent on changes in DNA sequences but rather result from chromosomal alterations. The transmission of epigenetic modifications through mitosis, meiosis, and transgenerational inheritance maintains homeostasis in multicellular organisms (Trerotola et al., 2015). While DNA methylation is a stable form of modification inherited by offspring (Margueron and Reinberg, 2010), histone acetylation modifications appear to be heritable based on studies demonstrating their persistence and activity in yeast cells over generations (Ekwall et al., 1997). However, the heritability of histone acetylation modifications in skeletal muscle has not been established. Genetic information related to skeletal muscle metabolism is transmitted through germ cells, and imprinted genes undergo epigenetic modifications during the transfer from parents to offspring, potentially influencing the next-generation’s gene expression patterns (Tucci et al., 2019). Notably, the maternally expressed imprinted gene H19, which encodes the lncRNA H19, plays a role in skeletal muscle satellite cell differentiation and insulin sensitivity mediated by SIRT1 and HDAC6 (Kumar and Datta, 2022). Maternal exercise before and during pregnancy has also been shown to mitigate insulin resistance in the skeletal muscle of female offspring from obese fathers (Falcao-Tebas et al., 2020). These findings suggest the possibility of transmitting information about histone acetylation modifications in skeletal muscle metabolism to future generations.

## 5 Summary and prospect

This review presents potential mechanisms underlying histone acetylation modifications in regulating skeletal muscle metabolism. It highlights the crucial roles of histone acetyltransferases (HATs) and histone deacetylases (HDACs) in maintaining skeletal muscle integrity, development, and insulin sensitivity. Furthermore, it suggests that enzymes involved in histone acetylation modifications could serve as potential therapeutic targets for certain muscle diseases. However, given the complexity of the organism, it is important to explore additional mechanisms by which histone acetylation modifications mediate skeletal muscle metabolism. The advancements in epigenomic sequencing technologies, such as ChIP-seq, CUT&Tag, ATAC-seq, Single-cell sequencing, and Spatial Transcriptomics, have provided powerful tools to investigate histone modifications and their impact on gene regulation in multiple dimensions. These technologies enable the study of cellular heterogeneity, intercellular communication, chromatin plasticity, and the precise regulation of histone acetylation modifications on phenotype. They also offer valuable insights for animal production and human health research.
